# Relationship between Cortical Thickness and EEG Alterations during Sleep in the Alzheimer’s Disease

**DOI:** 10.3390/brainsci11091174

**Published:** 2021-09-04

**Authors:** Aurora D’Atri, Maurizio Gorgoni, Serena Scarpelli, Susanna Cordone, Valentina Alfonsi, Camillo Marra, Michele Ferrara, Paolo Maria Rossini, Luigi De Gennaro

**Affiliations:** 1 Department of Biotechnological and Applied Clinical Sciences, University of L’Aquila, 67100 L’Aquila, Italy; aurora.datri@univaq.it (A.D.); michele.ferrara@univaq.it (M.F.); 2 Department of Psychology, University of Rome “Sapienza”, 00185 Rome, Italy; maurizio.gorgoni@uniroma1.it (M.G.); serena.scarpelli@uniroma1.it (S.S.); valentina.alfonsi@uniroma1.it (V.A.); 3 UniCamillus, Saint Camillus International University of Health Sciences, 00131 Rome, Italy; susanna.cordone@unicamillus.org; 4 Memory Clinic-Department of Aging, Neuroscience, Orthopaedic and Head-Neck, IRCCS Foundation Policlinico Universitario Agostino Gemelli, 00168 Rome, Italy; camillo.marra@policlinicogemelli.it; 5 Department of Neuroscience & Neurorehabil., IRCCS San Raffaele-Pisana, 00163 Rome, Italy; paolomaria.rossini@sanraffaele.it

**Keywords:** Alzheimer’s disease, sleep EEG, cortical thickness

## Abstract

Recent evidence showed that EEG activity alterations that occur during sleep are associated with structural, age-related, changes in healthy aging brains, and predict age-related decline in memory performance. Alzheimer’s disease (AD) patients show specific EEG alterations during sleep associated with cognitive decline, including reduced sleep spindles during NREM sleep and EEG slowing during REM sleep. We investigated the relationship between these EEG sleep alterations and brain structure changes in a study of 23 AD patients who underwent polysomnographic recording of their undisturbed sleep and 1.5T MRI scans. Cortical thickness measures were correlated with EEG power in the sigma band during NREM sleep and with delta- and beta-power during REM sleep. Thinning in the right precuneus correlated with all the EEG indexes considered in this study. Frontal–central NREM sigma power showed an inverse correlation with thinning of the left entorhinal cortex. Increased delta activity at the frontopolar and temporal regions was significantly associated with atrophy in some temporal, parietal, and frontal cortices, and with mean thickness of the right hemisphere. Our findings revealed an association between sleep EEG alterations and the changes to AD patients’ brain structures. Findings also highlight possible compensatory processes involving the sources of frontal–central sleep spindles.

## 1. Introduction

In recent years, the research on Alzheimer’s disease (AD) has seen growing interest in the alterations of sleep physiology and their possible role in the progression of the disease. In general, sleep seems to be a privileged physiological state for beta-amyloid clearance [1,2], while waking hours are characterized by progressive beta-amyloid deposition as a function of the time spent awake [3,4,5]. Sleep disturbances affect many patients with AD from the prodromal stage onwards [6,7,8,9]. Therefore, it has been hypothesized that the initial neurodegenerative processes of AD could alter sleep dynamics and that sleep disruptions could, in turn, increase beta-amyloid burden and establish a vicious circle that negatively impacts the neurodegenerative process [10].

Some quantitative EEG studies have described the alterations of cortical activity observed in sleeping AD patients. One of the most solid findings for NREM sleep in AD is the reduction in activity in the sigma frequency band (12–15 Hz) at parietal regions that likely mirrors the loss of sleep spindles [11,12,13,14]. Sleep spindles are phasic phenomena characteristic of NREM sleep, generated in the thalamo-cortical system [11]. These fast phasic oscillations are involved in sleep-dependent memory consolidation processes and particularly in the corticalization of hippocampus-dependent memory traces [15]. Accordingly, sleep spindle density is correlated with sleep-dependent gain in declarative memory performance in both healthy aging [16] and AD-related cognitive impairment [12].

Cortical activity during REM sleep also shows regional alterations in AD patients, similar to those occurring during their waking hours [17,18,19]. AD patients show a significant slowing of EEG activity, that is, an increase in slow-frequency cortical activity with a concomitant reduction in the highest frequencies. This shift toward the low frequencies mainly affects the temporal regions, and the alteration is even clearer during REM sleep than wakefulness in studies comparing AD patients and healthy controls [19]. EEG slowing during REM sleep showed a stronger correlation with cognitive decline than both NREM sigma activity and diurnal EEG slowing [19]. Previous studies found that EEG slowing during waking hours shared a strong negative correlation with cerebral oxygen metabolism in AD patients [20] and a positive correlation with the presence of structural lesions in patients with brain tumours [21]. This could suggest an index of the cortical damage in AD patients.

In recent years, some evidence has supported a correlation between specific EEG indexes of cortical activity during sleep and measures of cortical thickness in healthy young, and older, subjects [22,23,24]. Most of these studies focused on slow-wave activity (SWA) during NREM sleep [22,23] since this is the sleep EEG index characterized by the most evident changes across the lifespan [25]. These studies found that age-related reductions in SWA were strongly associated with cortical thinning and the volume of grey matter in the cortical generators of slow-wave activity, i.e., the frontal and prefrontal brain regions [26]. Delta activity, occurring at temporo-parieto-occipital regions during REM sleep, has similarly been shown to correlate with the integrity of frontal and prefrontal regions [24]. These findings support the idea that structural brain changes can alter EEG activity during sleep and impair some of the critical functions that sleep performs in daily life.

Moving from this evidence, we investigated the relationship between cortical thickness, measured using magnetic resonance imaging (MRI), and the major EEG alterations during NREM and REM sleep in AD patients [19], i.e., sigma EEG power during NREM sleep reflecting impaired spindle activity, and delta and beta power during REM sleep as indexes of the EEG slowing phenomenon.

Given the functional role of sleep spindles in the memory consolidation processes, we expected sigma activity during NREM sleep to reflect the compromise of its cortical generators in the precuneus [27], as well as those of memory-related cortical areas significantly impaired in AD. On the other hand, EEG slowing during REM sleep, i.e., increased cortical activity at the slowest frequencies and reduced activity at the fastest ones, was expected to show a less specific and more generalized relationship with the structural changes of the neurodegenerative AD process.

## 2. Materials and Methods

### 2.1. Participants

The 23 (out of 50) subjects included in the study by D’Atri et al. [19] reporting a diagnosis of Alzheimer’s disease (AD) with available MR data were included in the current study (12 females: mean age 73.2 y ± s. d. 6.2 y).

Details on the diagnostic procedure and inclusion/exclusion criteria are reported elsewhere [19]. Briefly, the AD diagnosis conformed to the DSM-IV and the National Institute on Aging–Alzheimer’s Association workgroup’s [28] criteria (MMSE mean score 19.0 ± s. d. 4.3). The presence of neurological, psychiatric, or vascular disorders, diagnosed sleep disorders, histories of seizures, psychoactive/hypnotic drug use, and history of alcoholism or drug abuse were exclusion criteria.

All participants (or their caregivers) gave written informed consent to participate in the study. The study was performed in accordance with the declaration of Helsinki and was approved by the Local Ethics Committee.

### 2.2. Polysomnographic Recordings

Subjects underwent a standard polysomnography (PSG) during a night of undisturbed sleep in a video-monitored, soundproof, and electrically shaded room at the Policlinico Universitario A. Gemelli. PSG signals included 19 cortical EEG derivations of the international standard 10–20 system, A1 and A2 at the mastoids, EOGs and submental EMGs, recorded via a Micromed Morpheus digital polygraph (Micromed, Mogliano Veneto, Treviso, Italy). EEG signals from the 19 cortical sites were re-referenced offline to the averaged mastoids and filtered between 0.33 and 30 Hz (for details, see [19]).

### 2.3. Sleep EEG Analysis

Sleep macrostructure was obtained by visually scoring PSG recordings (20 s epoch duration) according to the AASM criteria [29]. The following macrostructural variables were computed: sleep onset (SO) latency (defined by the first K-complex or >0.5 s sleep spindle); REM latency; duration of N1, N2, N3, and REM sleep stages (%); duration of wakefulness after SO (WASO); number of awakenings; total sleep time (TST; as the sum of time spent in N1, N2, N3, and REM); total bed time (TBT); sleep efficiency index (total sleep time/total bed time × 100).

Spectral power from each cortical site was computed separately for NREM (including N2 and N3) and REM sleep by considering the whole recording and through Fast Fourier Transform (FFT) of the artefact-free epoch (4 s periodogram) in the 0.50–30.00 Hz interval (0.25 Hz bin resolution).

In order to control for possible between-subject differences in the DC-offset raw power spectra maintaining the spatial distribution of power, we computed the percentage power (%) of each frequency bin using the total power in the frequency range of interest (0.50–25.00 Hz), considering the whole topography (19 cortical sites) in further analyses. The spectral power in the standard frequency bands for each cortical site were calculated as the sum of the percentage power for the corresponding frequency bins (delta: 0.50–4.75 Hz; theta: 5.00–7.75 Hz; alpha: 8.00–11.75 Hz; sigma: 12.00–15.75 Hz; beta: 16:00–24.75 Hz). The topographic distributions of the mean power (%) in the 5 frequency bands for NREM and REM sleep are reported in Supplemental Figure S1.

**Figure 1:**
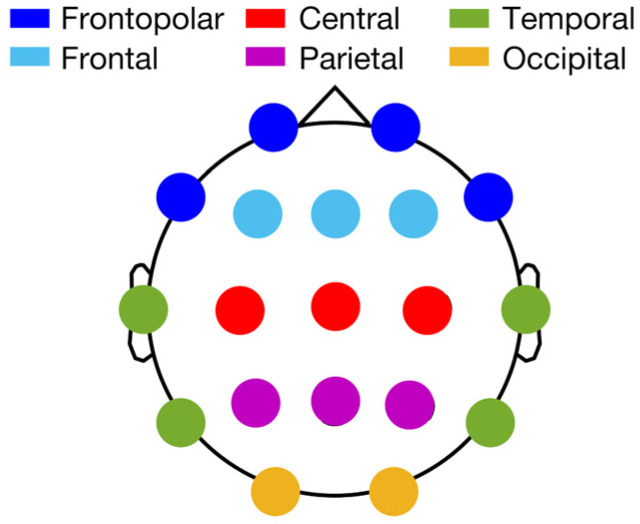
EEG clusters for correlational analysis with MR data, with EEG electrodes subdivided into 6 topographical clusters according to their location on the scalp. Frontopolar cluster: Fp1, Fp2, F7, F8 (dark blue); frontal cluster: F3, F4, Fz (light blue); central cluster: C3, C4, Cz (red), parietal cluster: P3, P4, Pz (purple); temporal cluster: T3, T4, T5, T6 (green); occipital cluster: O1, O2 (yellow).

In order to reduce the number of correlations with the MR data, we adopted a by-cluster approach to the sleep EEG data considering 6 clusters covering the whole cortical topography (Figure 1): frontopolar (Fp1, Fp2, F7, F8), frontal (F3, F4, Fz), central (C3, C4, Cz), parietal (P3, P4, Pz), temporal (T3, T4, T5, T6), and occipital (O1, O2). The EEG power of each cluster was computed as the sum of the power (%) of the included derivations.

### 2.4. MRI Acquisition and Processing

MRI sessions were acquired on a 1.5T scanner (Signa, GE Healthcare) and included a 3D T1 MPRAGE scan (TR = 2000 ms, TE = 3.42 ms, flip angle = 15 degrees, FOV = 256 mm, 1.0 mm slice thickness for a total of 160 slices per slab, matrix size = 256, NEX = 1).

Anatomical, high-resolution images (3D T1) were segmented and parcellated using FreeSurfer’s recon-all standard pipeline [30,31]. Briefly, after brain extraction, high-resolution 3D T1 images were registered to standard space and the grey/white and grey/cerebrospinal fluid borders were computed [30,32,33]. An experienced neuroradiologist and imaging analyst (blind to clinical data) visually inspected the image outputs from each stage of FreeSurfer processing and edited them to refine segmentation and correct any software delineation errors. Cortical parcellations were made according to Desikan atlas [34] and thickness measurements of 34 cortical regions (bank of the superior temporal sulcus, caudal anterior cingulate, caudal middle frontal, cuneus, entorhinal, fusiform, inferior parietal, inferior temporal, isthmus cingulate, lateral occipital, lateral orbitofrontal, lingual, medial orbitofrontal, middle temporal, parahippocampal, paracentral, pars opercularis, pars orbitalis, pars triangularis, pericalcarine, postcentral, posterior cingulate, precentral, precuneus, rostral anterior cingulate, rostral middle frontal, superior frontal, superior parietal, superior temporal, supramarginal, frontal pole, temporal pole, transverse temporal, and insula). Hemispheric mean thickness measurements were collected from both the right and left hemispheres.

### 2.5. Statistical Analyses

Statistical analyses were performed using MATLAB (R2020b).

We focused our analyses on the EEG indexes that better characterize sleep in AD as compared to healthy controls, i.e., the sigma power for NREM sleep and the delta- and beta-power for REM sleep. These sleep EEG indexes, computed using the 6 EEG clusters, were correlated (Pearson’s r) with the cortical thickness of areas identified according to the Desikan atlas in the two hemispheres. For each cortical area of the MR data, we controlled for multiple comparisons using the false discovery rate method (FDR [35]) considering the correlations with NREM and REM sleep indexes in the 6 EEG clusters. This occurred separately for left and right hemispheres (FDR-adjusted *p* ≤ 0.05).

A control analysis using partial correlations was performed on significant correlations to control for the possible confounding effects of age and sex.

## 3. Results

Table 1 reports the descriptive statistics of sleep macrostructure from the PSG recordings.

The statistically significant results of the correlational analyses between cortical thickness and EEG activity during NREM and REM sleep in AD are reported in Table 2.

**Figure 2:**
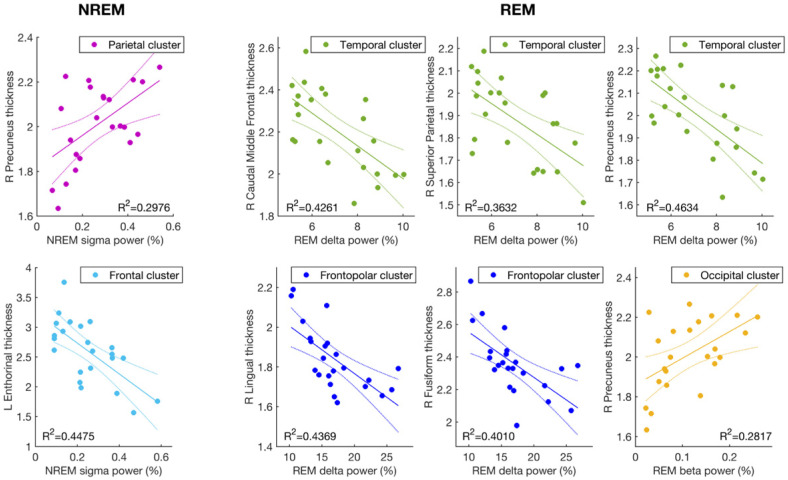
Correlations between cortical thickness and sleep EEG indexes in AD patients. Cortical thickness (mm) vs. EEG power (%) scatterplots for cortical areas and sleep EEG indexes show a significant relationship after FDR correction. When the thickness of an area was significantly correlated with two or more EEG clusters for the same sleep index, only data of the EEG cluster reporting the highest correlation are shown (see Supplemental Figure S2 for the others). R^2^ coefficients of the linear regressions are also reported.

Regional NREM and REM EEG indexes were significantly correlated to the cortical thickness of different temporal, parietal, and frontal regions. Figure 2 shows the strongest cortical thickness vs. EEG power correlation for each sleep EEG index (see Supplemental Figure S1 for other significant correlations).

### 3.1. Cortical Thickness and Sigma Activity of NREM Sleep

NREM sigma power of the parietal EEG cluster was positively correlated to cortical thickness in the right precuneus cortex (r = 0.55; *p* = 0.0071), while the anterior clusters (frontopolar, frontal, and central) showed a robust negative relationship with the thickness of the left entorhinal cortex (r ≤ −0.62; *p* ≤ 0.0017); i.e., higher sigma power was associated with lower entorhinal cortex thickness.

### 3.2. Cortical Thickness and the EEG Slowing (Delta and Beta Activity) during REM Sleep

Delta REM activity at the frontopolar and temporal clusters was significantly correlated to cortical atrophy in the left and right caudal middle frontal gyri, the right fusiform and lingual gyri, the precuneus, and the superior parietal cortices (r ≤ −0.58; *p* ≤ 0.0035). More generally, and consistent with the findings of EEG slowing during REM sleep as a sign of AD, frontopolar and temporal delta activity during REM sleep was inversely related to the mean thickness of the whole right hemisphere (r ≤ −0.58; *p* ≤ 0.0035), so that subjects with higher cortical atrophy (i.e., lesser thickness) also showed higher delta power in the frontotemporal regions during REM sleep.

High-frequency beta activity, the counterpart of REM EEG slowing, also showed a significant relationship with the right precuneus cortex at the temporal and occipital EEG clusters but in the opposite direction (r ≥ 0.52, *p* ≤ 0.012), with lower beta power associated with lower cortical thickness.

The results of the analyses controlling for age and sex confirmed all these findings, as reported in Supplemental Table S1.

## 4. Discussion

Findings from the present study confirm the relationship between alterations of the EEG activity during sleep and the structural changes to the brains of AD patients. In particular, the cortical thickness of the caudal middle frontal gyrus, entorhinal cortex, fusiform gyrus, lingual gyrus, precuneus cortex, and superior parietal cortex, showed significant correlations with the sleep EEG indexes under consideration. The neurodegenerative process of AD affects all of these cortical areas in the course of the disease [36], leading to worsening symptoms as the disease progresses. The entorhinal cortex and fusiform gyrus areas of the temporal lobe undergo early, and the most severe, atrophy [37,38]. Damage to these areas is linked to the early memory impairment affecting these patients even some years before the diagnosis of AD [39]. The superior parietal cortex and the precuneus also show grey matter loss before the progression of mild cognitive impairment to AD [40,41,42,43,44,45,46,47,48]. Functional and structural alterations in these areas have been associated with cognitive dysfunctions, particularly in autobiographical memory retrieval [49,50]. Finally, the thinning of the caudal middle frontal cortex has been linked to executive dysfunctions, including in the attention domain [51].

As expected, we found that parietal sigma activity during NREM is related to the cortical thickness of the precuneus, one of the main cortical generators of sleep spindles [27]. This finding suggests that the reduction in parietal sleep spindles that has been described in AD patients [12] is linked to the impairment of the cortical sources of this sleep rhythm. Unexpectedly, frontal and central sigma activity showed a strong negative relationship with the cortical thickness of the left entorhinal cortex. This part of the medial temporal lobe plays an essential role in declarative memory [52], representing the main source of inputs for the hippocampus, and it is one of the regions damaged earliest, and most severely, by AD [37]. Given the role of sleep spindles in memory consolidation processes, we expected spindle activity to be impaired due to neurodegeneration of memory-related areas. However, unlike parietal spindles, frontal sleep spindles have cortical generators located in the frontal cortex (Brodmann areas 9 and 10) [27,53]. Accordingly, the neurodegenerative process in AD could impact less heavily on anterior cortical sources of sleep spindles than on posterior ones. Consistently, only fast parietal spindles have been reported to be impaired in these patients [12]. Along this line, a speculative interpretation of the negative relationship between centro-frontal sigma power and the thickness of the entorhinal cortex in AD could involve a mechanism of neural compensation in sleep-dependent memory consolidation processes.

The activation of compensatory pathways in brain functional connectivity during memory tasks following damage to the medial temporal lobe has been reported in AD [54,55]. It is possible that the remaining ability to consolidate memory traces at the cortical level via sleep spindles follows similar patterns, and preferentially involves brain regions less affected by the disease’s neurodegenerative processes. From this viewpoint, the inverse relationship between spindle activity (of the frontal and central areas) and entorhinal cortex atrophy could reflect the signature of neural compensatory mechanisms by which the brain affected by AD tries to overcome the functional changes related to neurodegeneration during waking hours. However, the lack of similar measurements on healthy control subjects in the current study and in the scientific literature does not allow us to exclude the presence of the same relationship in healthy aging populations.

Results of the REM sleep EEG indexes confirm a strong relationship between EEG slowing and cortical atrophy in different districts of the brain. Delta activity at frontotemporal regions during REM sleep is significantly correlated to the mean thickness of the right hemisphere and, therefore, might be considered an EEG index of the neurodegenerative process in brains affected by AD. Specifically, both delta and beta REM activity show strong correlations, in opposite directions, with the thickness of the precuneus. Delta EEG activity of the temporal areas also negatively correlates with thinning of the right superior parietal cortex and bilateral caudal middle frontal cortex. Moreover, the increase in slow-frequency activity at frontopolar and temporal regions negatively correlates with the thickness of the fusiform and lingual cortices in the medial temporal lobe. The different directions of the correlation with cortical thickness among NREM sigma and REM delta activity is possibly explained by the different origins of these EEG alterations. In AD cases, parietal sigma activity reduces with increasing atrophy of the precuneus, probably because it is the main cortical generator of this EEG rhythm [27]. The increase in slow-frequency activity in the EEG slowing during REM sleep likely does not depend on alterations to the cortical generators of REM delta activity, but rather, could be considered a secondary effect of the loss of neurons and connections due to the progression of the disease. Indeed, cortical atrophy in AD progression follows a temporo-parieto-frontal trajectory, with the medial temporal lobe showing a marked neurodegeneration in early stages, while motor and sensory cortices remain quite preserved until the latest stages of the disease (for a review, see [56]). The cortical generators of delta waves during REM sleep have been recently localized in the occipital cortex and in the medial frontal area (the midcingulate gyrus, supplementary motor area, and medial part of the primary motor cortex) [57] and so they should be less affected by neurodegeneration in AD patients. As mentioned above, EEG slowing also characterizes AD patients in their waking hours [19,58,59] and patients with brain injuries, especially in the areas surrounding the lesions [60]. Findings from experimental studies suggest that the occurrence of slow waves in the EEG could be a consequence of the deafferentation of cortical regions [61]. A recent TMS/EEG study described this as a functional consequence of focal brain injury and stroke in perilesional areas [62]. In the study, areas adjacent to (but not directly involved with) the lesion showed a sleep-like response to the local TMS stimulation during waking hours [62]. The authors suggested that alterations in the balance between excitatory and inhibitory inputs from the nearby damaged region mimic a state of physiological sleep and drive the perilesional zone to show local, sleep-like activity during waking hours [62].

From the EEG point of view, REM sleep is closer to the waking state than to NREM sleep, since it is characterized by high-frequency and desynchronized activity as opposed to synchronized SWA that is peculiar in deepest NREM sleep. The strong relationship between the increase in slow-frequency EEG activity in frontopolar and temporal regions during REM sleep, and the thinning of cortical areas along the trajectory of the neurodegenerative process of AD supports the theory of EEG slowing as a consequence of cortical deafferentation due to the progressive loss of neurons. In this sense, it is interesting to note that slow-frequency activity during REM sleep at the temporal regions shows a correlation with caudal middle frontal cortex atrophy that is even stronger than correlations with cortices in the temporal lobe, while frontopolar delta activity shows very strong associations with the thinning of temporo-parietal cortices, but no significant relationship with thinning in anterior areas. The strengthening of the relationship as distance increases further supports the theory of impairment in long-distance connections in areas most affected by EEG slowing due to AD neurodegeneration.

Two main limitations affect the findings of the current study. Firstly, the small sample size means that great caution is needed when interpreting our results and that future replications in studies involving larger numbers of patients is needed. Secondly, the lack of a direct comparison with healthy aging individuals means that we cannot exclude the possibility that some of the relationships highlighted here could be linked to age-related brain changes rather than AD-related neurodegenerative processes. Our supplemental analyses controlling for age and sex, however, showed that these relationships remained significant even after excluding the specific contribution of age and sex.

## 5. Conclusions

This is the first investigation on the relationship among EEG activity during AD patients’ sleep and measures of AD patients’ cortical thickness. We found that reductions in parietal spindle activity (12–15 Hz) were related to atrophy of the precuneus, one of its cortical sources, while frontal–central NREM sigma activity exhibits an inverse relationship with the cortical thickness of the entorhinal cortex. This suggests that compensatory mechanisms in sleep-dependent memory consolidation processes within these brain regions are occurring. Moreover, we showed that the increase in slow-frequency activity in frontotemporal regions during REM sleep correlates with atrophy in some brain areas most affected during the course of the disease. This finding supports EEG slowing as a promising index of progressive neurodegeneration within the brains of AD patients.

## Figures and Tables

**Table 1:** Sleep macrostructure. Descriptive statistics (means and standard deviations) of the macrostructural variables of sleep from the PSG recordings in the AD sample.

PSG Measure ^1^	Mean	s.d.
SO latency (min)	26.60	19.99
REM latency (min)	87.03	49.04
WASO (min)	74.71	44.46
N1 (%)	7.05	6.37
N2 (%)	74.33	6.95
N3 (%)	0.56	1.28
REM (%)	18.24	6.81
TBT (min)	373.58	70.16
TST (min)	268.87	76.22
SEI (%)	71.40	13.00
ISA (#)	19.74	12.14

^1^ Abbreviations: AD = Alzheimer’s disease; ISA = intra-sleep awakenings; REM = rapid eye movement; N1 = NREM 1 Stage; N2 = NREM 2 Stage; N3 = NREM 3 Stage; s.d. = standard deviation; SEI = sleep efficiency index; SO = sleep onset; TBT = total bed time; TST = total sleep time; WASO = wake after sleep onset.

**Table 2:** Relationships between cortical thickness and sleep EEG alterations in AD. Significant (FDR-adjusted *p* ≤ 0.05) correlations between cortical thickness and sleep EEG alterations (NREM sigma power, REM delta and beta power) during sleep in AD. Pearson’s *r* coefficients and corresponding uncorrected *p* values are reported.

MR Cortical Areas	Side	Sleep Index	EEG Cluster	r	*p*
Caudal middle frontal	L	REM Delta	Temporal	−0.62	0.0016
	R	REM Delta	Temporal	−0.65	0.00073
Entorhinal	L	NREM sigma	Frontopolar	−0.62	0.0017
	L	NREM sigma	Frontal	−0.67	0.00048
	L	NREM sigma	Central	−0.62	0.0017
Fusiform	R	REM Delta	Frontopolar	−0.63	0.00122
	R	REM Delta	Temporal	−0.58	0.0035
Lingual	R	REM Delta	Frontopolar	−0.66	0.00060
	R	REM Delta	Temporal	−0.60	0.0027
Precuneus	R	NREM sigma	Parietal	0.55	0.0071
	R	REM delta	Frontopolar	−0.62	0.0017
	R	REM delta	Temporal	−0.68	0.00035
	R	REM beta	Temporal	0.52	0.012
	R	REM beta	Occipital	0.53	0.0092
Superior parietal	R	REM delta	Temporal	−0.60	0.0023
Mean thickness	R	REM delta	Frontopolar	−0.60	0.0027
	R	REM delta	Temporal	−0.59	0.0028

Abbreviations: MR, magnetic resonance; L, left hemisphere; R, right hemisphere; NREM, non-rapid eye movement sleep; REM, rapid eye movement sleep.

## Data Availability

The data presented in this study are available on request from the corresponding author.

## Supplementary Materials

The following are available online at https://www.mdpi.com/article/10.3390/brainsci11091174/s1, Figure S1: topographic distribution of EEG power (%) during NREM and REM sleep in the sample of AD patients, Figure S2: correlations between cortical thickness and sleep EEG indexes, Table S1: results from the control analysis with partial correlations (Pearson’s r and corresponding *p* value) controlling for age and sex on cortical thickness and relevant EEG indexes of NREM and REM sleep showing significant associations in the principal analysis.

## References

[1] Kang J.-E., Lim M.M., Bateman R.J., Lee J.J., Smyth L.P., Cirrito J.R., Fujiki N., Nishino S., Holtzman D.M. (2009). Amyloid- Dynamics Are Regulated by Orexin and the Sleep-Wake Cycle. Science.

[2] Xie L., Kang H., Xu Q., Chen M.J., Liao Y., Thiyagarajan M., O’Donnell J., Christensen D.J., Nicholson C., Iliff J.J. (2013). Sleep Drives Metabolite Clearance from the Adult Brain. Science.

[3] Ju Y.-E.S., Ooms S.J., Sutphen C., Macauley S.L., Zangrilli M.A., Jerome G., Fagan A.M., Mignot E., Zempel J.M., Claassen J.A.H.R. (2017). Slow wave sleep disruption increases cerebrospinal fluid amyloid-β levels. Brain.

[4] Lucey B.P., Hicks T.J., McLeland J.S., Toedebusch C.D., Boyd J., Elbert D.L., Patterson B.W., Baty J., Morris J.C., Ovod V. (2018). Effect of sleep on overnight cerebrospinal fluid amyloid β kinetics. Ann. Neurol..

[5] Ooms S., Overeem S., Besse K., Rikkert M.O., Verbeek M., Claassen J.A.H.R. (2014). Effect of 1 Night of Total Sleep Deprivation on Cerebrospinal Fluid β-Amyloid 42 in Healthy Middle-Aged Men. JAMA Neurol..

[6] Prinz P.N., Vitaliano P.P., Vitiello M.V., Bokan J., Raskind M., Peskind E., Gerber C. (1982). Sleep, EEG and mental function changes in senile dementia of the Alzheimer’s type. Neurobiol. Aging.

[7] Vitiello M.V., Prinz P.N., Williams D.E., Frommlet M.S., Ries R.K. (1990). Sleep Disturbances in Patients With Mild-Stage Alzheimer’s Disease. J. Gerontol..

[8] Moe K.E., Vitiello M.V., Larsen L.H., Prinz P.N. (1995). Sleep/wake patterns In Alzheimer’s disease: Relationships with cognition and function. J. Sleep Res..

[9] Cordone S., Scarpelli S., Alfonsi V., De Gennaro L., Gorgoni M. (2021). Sleep-Based Interventions in Alzheimer’s Disease: Promising Approaches from Prevention to Treatment along the Disease Trajectory. Pharmaceuticals.

[10] Lim M.M., Gerstner J.R., Holtzman D.M. (2014). The sleep-wake cycle and Alzheimer’s disease: What do we know?. Neurodegener. Dis. Manag..

[11] De Gennaro L., Ferrara M. (2003). Sleep spindles: An overview. Sleep Med..

[12] Gorgoni M., Lauri G., Truglia I., Cordone S., Sarasso S., Scarpelli S., Mangiaruga A., D’Atri A., Tempesta D., Ferrara M. (2016). Parietal Fast Sleep Spindle Density Decrease in Alzheimer’ s Disease and Amnesic Mild Cognitive Impairment. Neural Plast..

[13] Rauchs G., Schabus M., Parapatics S., Bertran F., Clochon P., Hot P., Denise P., Desgranges B., Eustache F., Gruber G. (2008). Is there a link between sleep changes and memory in Alzheimer’s disease?. Neuroreport.

[14] Westerberg C.E., Mander B.A., Florczak S.M., Weintraub S., Mesulam M.-M., Zee P.C., Paller K.A. (2012). Concurrent Impairments in Sleep and Memory in Amnestic Mild Cognitive Impairment. J. Int. Neuropsychol. Soc..

[15] Klinzing J.G., Niethard N., Born J. (2019). Mechanisms of systems memory consolidation during sleep. Nat. Neurosci..

[16] Mander B.A., Rao V., Lu B., Saletin J.M., Ancoli-Israel S., Jagust W.J., Walker M.P. (2014). Impaired prefrontal sleep spindle regulation of hippocampal-dependent learning in older adults. Cereb. Cortex.

[17] Brayet P., Petit D., Frauscher B., Gagnon J.-F., Gosselin N., Gagnon K., Rouleau I., Montplaisir J. (2016). Quantitative EEG of Rapid-Eye-Movement Sleep: A Marker of Amnestic Mild Cognitive Impairment. Clin. EEG Neurosci..

[18] Hassainia F., Petit D., Nielsen T., Gauthier S., Montplaisir J. (1997). Quantitative EEG and Statistical Mapping of Wakefulness and REM Sleep in the Evaluation of Mild to Moderate Alzheimer’s Disease. Eur. Neurol..

[19] D’Atri A., Scarpelli S., Gorgoni M., Truglia I., Lauri G., Cordone S., Ferrara M., Marra C., Rossini P.M., De Gennaro L. (2021). EEG alterations during wake and sleep in mild cognitive impairment and Alzheimer’s disease. iScience.

[20] Buchan R.J., Nagata K., Yokoyama E., Langman P., Yuya H., Hirata Y., Hatazawa J., Kanno I. (1997). Regional correlations between the EEG and oxygen metabolism in dementia of Alzheimer’s type. Electroencephalogr. Clin. Neurophysiol..

[21] Nagata K., Gross C.E., Kindt G.W., Geier J.M., Adey G.R. (1985). Topographic Electroencephalographic Study with Power Ratio Index Mapping in Patients with Malignant Brain Tumors. Neurosurgery.

[22] Dubé J., Lafortune M., Bedetti C., Bouchard M., Gagnon J.F., Doyon J., Evans A.C., Lina J.-M., Carrier J. (2015). Cortical thinning explains changes in sleep slow waves during adulthood. J. Neurosci..

[23] Mander B.A., Rao V., Lu B., Saletin J.M., Lindquist J.R., Ancoli-Israel S., Jagust W., Walker M.P. (2013). Prefrontal atrophy, disrupted NREM slow waves and impaired hippocampal-dependent memory in aging. Nat. Neurosci..

[24] Latreille V., Gaubert M., Dubé J., Lina J.-M., Gagnon J.-F., Carrier J. (2019). Age-related cortical signatures of human sleep electroencephalography. Neurobiol. Aging.

[25] Mander B.A., Winer J.R., Walker M.P. (2017). Sleep and Human Aging. Neuron.

[26] Mander B.A., Marks S.M., Vogel J.W., Rao V., Lu B., Saletin J.M., Ancoli-Israel S., Jagust W.J., Walker M.P. (2015). β-amyloid disrupts human NREM slow waves and related hippocampus-dependent memory consolidation. Nat. Neurosci..

[27] Anderer P., Klösch G., Gruber G., Trenker E., Pascual-Marqui R., Zeitlhofer J., Barbanoj M., Rappelsberger P., Saletu B. (2001). Low-resolution brain electromagnetic tomography revealed simultaneously active frontal and parietal sleep spindle sources in the human cortex. Neuroscience.

[28] McKhann G.M., Knopman D.S., Chertkow H., Hyman B.T., Jack C.R., Kawas C.H., Klunk W.E., Koroshetz W.J., Manly J.J., Mayeux R. (2011). The diagnosis of dementia due to Alzheimer’s disease: Recommendations from the National Institute on Aging-Alzheimer’s Association workgroups on diagnostic guidelines for Alzheimer’s disease. Alzheimer’s Dement..

[29] Iber C., Ancoli-Israel S., Chesson A., Quan S. (2007). The AASM Manual for the Scoring of Sleep and Associates Events: Rules, Terminology and Technical Specifications.

[30] Fischl B., Salat D.H., Busa E., Albert M., Dieterich M., Haselgrove C., van der Kouwe A., Killiany R., Kennedy D., Klaveness S. (2002). Whole Brain Segmentation. Neuron.

[31] Fischl B., Sereno M.I., Dale A.M. (1999). Cortical Surface-Based Analysis II: Inflation, flattening, and a surface-based coordinate system. Neuroimage.

[32] Dale A.M., Fischl B., Sereno M.I. (1999). Cortical Surface-Based Analysis I: Segmentation and surface reconstruction. Neuroimage.

[33] Fischl B., Sereno M.I., Tootell R.B.H., Dale A.M. (1999). High-resolution intersubject averaging and a coordinate system for the cortical surface. Hum. Brain Mapp..

[34] Desikan R.S., Ségonne F., Fischl B., Quinn B.T., Dickerson B.C., Blacker D., Buckner R.L., Dale A.M., Maguire R.P., Hyman B.T. (2006). An automated labeling system for subdividing the human cerebral cortex on MRI scans into gyral based regions of interest. Neuroimage.

[35] Benjamini Y., Hochberg Y. (1995). Controlling the False Discovery Rate: A Practical and Powerful Approach to Multiple Testing. J. R. Stat. Soc. Ser. B.

[36] Yang H., Xu H., Li Q., Jin Y., Jiang W., Wang J., Wu Y., Li W., Yang C., Li X. (2019). Study of brain morphology change in Alzheimer’s disease and amnestic mild cognitive impairment compared with normal controls. Gen. Psychiatry.

[37] Van Hoesen G.W., Hyman B.T., Damasio A.R. (1991). Entorhinal cortex pathology in Alzheimer’s disease. Hippocampus.

[38] Whitwell J.L. (2010). Progression of Atrophy in Alzheimer’s Disease and Related Disorders. Neurotox. Res..

[39] Golby A., Silverberg G., Race E., Gabrieli S., O’Shea J., Knierim K., Stebbins G., Gabrieli J. (2005). Memory encoding in Alzheimer’s disease: An fMRI study of explicit and implicit memory. Brain.

[40] Bakkour A., Morris J.C., Dickerson B.C. (2009). The cortical signature of prodromal AD: Regional thinning predicts mild AD dementia. Neurology.

[41] Bozzali M., Filippi M., Magnani G., Cercignani M., Franceschi M., Schiatti E., Castiglioni S., Mossini R., Falautano M., Scotti G. (2006). The contribution of voxel-based morphometry in staging patients with mild cognitive impairment. Neurology.

[42] Chételat G., Landeau B., Eustache F., Mézenge F., Viader F., de la Sayette V., Desgranges B., Baron J.-C. (2005). Using voxel-based morphometry to map the structural changes associated with rapid conversion in MCI: A longitudinal MRI study. Neuroimage.

[43] Desikan R.S., Fischl B., Cabral H.J., Kemper T.L., Guttmann C.R.G., Blacker D., Hyman B.T., Albert M.S., Killiany R.J. (2008). MRI measures of temporoparietal regions show differential rates of atrophy during prodromal AD. Neurology.

[44] Desikan R.S., Cabral H.J., Fischl B., Guttmann C.R.G., Blacker D., Hyman B.T., Albert M.S., Killiany R.J. (2009). Temporoparietal MR Imaging Measures of Atrophy in Subjects with Mild Cognitive Impairment That Predict Subsequent Diagnosis of Alzheimer Disease. Am. J. Neuroradiol..

[45] Hämäläinen A., Tervo S., Grau-Olivares M., Niskanen E., Pennanen C., Huuskonen J., Kivipelto M., Hänninen T., Tapiola M., Vanhanen M. (2007). Voxel-based morphometry to detect brain atrophy in progressive mild cognitive impairment. Neuroimage.

[46] Julkunen V., Niskanen E., Muehlboeck S., Pihlajamäki M., Könönen M., Hallikainen M., Kivipelto M., Tervo S., Vanninen R., Evans A. (2009). Cortical Thickness Analysis to Detect Progressive Mild Cognitive Impairment: A Reference to Alzheimer’s Disease. Dement. Geriatr. Cogn. Disord..

[47] Karas G., Sluimer J., Goekoop R., van der Flier W., Rombouts S.A.R.B., Vrenken H., Scheltens P., Fox N., Barkhof F. (2008). Amnestic Mild Cognitive Impairment: Structural MR Imaging Findings Predictive of Conversion to Alzheimer Disease. Am. J. Neuroradiol..

[48] Whitwell J.L., Shiung M.M., Przybelski S.A., Weigand S.D., Knopman D.S., Boeve B.F., Petersen R.C., Jack C.R. (2008). MRI patterns of atrophy associated with progression to AD in amnestic mild cognitive impairment. Neurology.

[49] Buckner R.L. (2005). Molecular, Structural, and Functional Characterization of Alzheimer’s Disease: Evidence for a Relationship between Default Activity, Amyloid, and Memory. J. Neurosci..

[50] Jack C.R., Knopman D.S., Jagust W.J., Shaw L.M., Aisen P.S., Weiner M.W., Petersen R.C., Trojanowski J.Q. (2010). Hypothetical model of dynamic biomarkers of the Alzheimer’s pathological cascade. Lancet Neurol..

[51] Grambaite R., Selnes P., Reinvang I., Aarsland D., Hessen E., Gjerstad L., Fladby T. (2011). Executive Dysfunction in Mild Cognitive Impairment is Associated with Changes in Frontal and Cingulate White Matter Tracts. J. Alzheimer’s Dis..

[52] Squire L.R., Stark C.E.L., Clark R.E. (2004). The medial temporal lobe. Annu. Rev. Neurosci..

[53] Alfonsi V., D’Atri A., Gorgoni M., Scarpelli S., Mangiaruga A., Ferrara M., De Gennaro L. (2019). Spatiotemporal dynamics of sleep spindle sources across NREM sleep cycles. Front. Neurosci..

[54] Rosenbaum R.S., Furey M.L., Horwitz B., Grady C.L. (2010). Altered connectivity among emotion-related brain regions during short-term memory in Alzheimer’s disease. Neurobiol. Aging.

[55] Meulenbroek O., Rijpkema M., Kessels R.P.C., Rikkert M.G.M.O., Fernández G. (2010). Autobiographical memory retrieval in patients with Alzheimer’s disease. Neuroimage.

[56] Pini L., Pievani M., Bocchetta M., Altomare D., Bosco P., Cavedo E., Galluzzi S., Marizzoni M., Frisoni G.B. (2016). Brain atrophy in Alzheimer’s Disease and aging. Ageing Res. Rev..

[57] Bernardi G., Betta M., Ricciardi E., Pietrini P., Tononi G., Siclari F. (2019). Regional Delta Waves In Human Rapid Eye Movement Sleep. J. Neurosci..

[58] Babiloni C., Lizio R., Marzano N., Capotosto P., Soricelli A., Triggiani A.I., Cordone S., Gesualdo L., Del Percio C. (2015). Brain neural synchronization and functional coupling in Alzheimer’s disease as revealed by resting state EEG rhythms. Int. J. Psychophysiol..

[59] Jeong J. (2004). EEG dynamics in patients with Alzheimer’s disease. Clin. Neurophysiol..

[60] Nuwer M.R., Jordan S.E., Ahn S.S. (1987). Evaluation of stroke using EEG frequency analysis and topographic mapping. Neurology.

[61] Nita D.A., Cisse Y., Timofeev I., Steriade M. (2007). Waking-Sleep Modulation of Paroxysmal Activities Induced by Partial Cortical Deafferentation. Cereb. Cortex.

[62] Sarasso S., D’Ambrosio S., Fecchio M., Casarotto S., Viganò A., Landi C., Mattavelli G., Gosseries O., Quarenghi M., Laureys S. (2020). Local sleep-like cortical reactivity in the awake brain after focal injury. Brain.

