# Supplementary material for: Relationship between Cortical Thickness and EEG Alterations during Sleep in the Alzheimer’s Disease
Source: Brain Sci. 2021 Sep 4;11(9):1174. doi: 10.3390/brainsci11091174 (PMC8468220; doi:10.3390/brainsci11091174)
Supplement: Supplementary file 1 [file brainsci-11-01174-s001.zip › brainsci-1342224-supplementary.pdf]

Supplemental Materials

Supplemental Figures

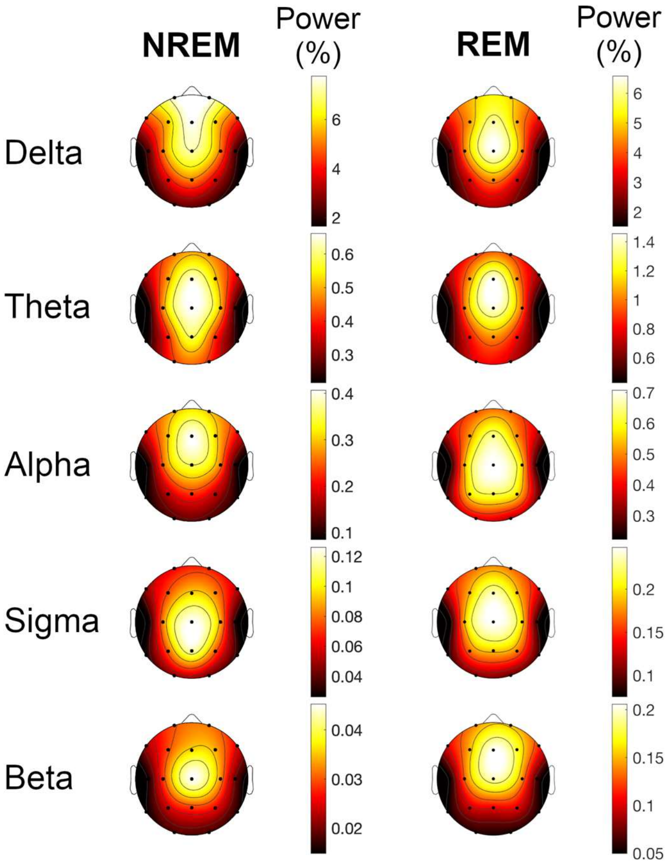

Figure S1. Topographic distribution of EEG power (%) during NREM and REM sleep in the sample of AD patients. Topographic maps of the spectral power (%) during NREM (1st column) and REM sleep (2nd column) in AD. The topographic maps are scaled between minimal and maximal values within each map.

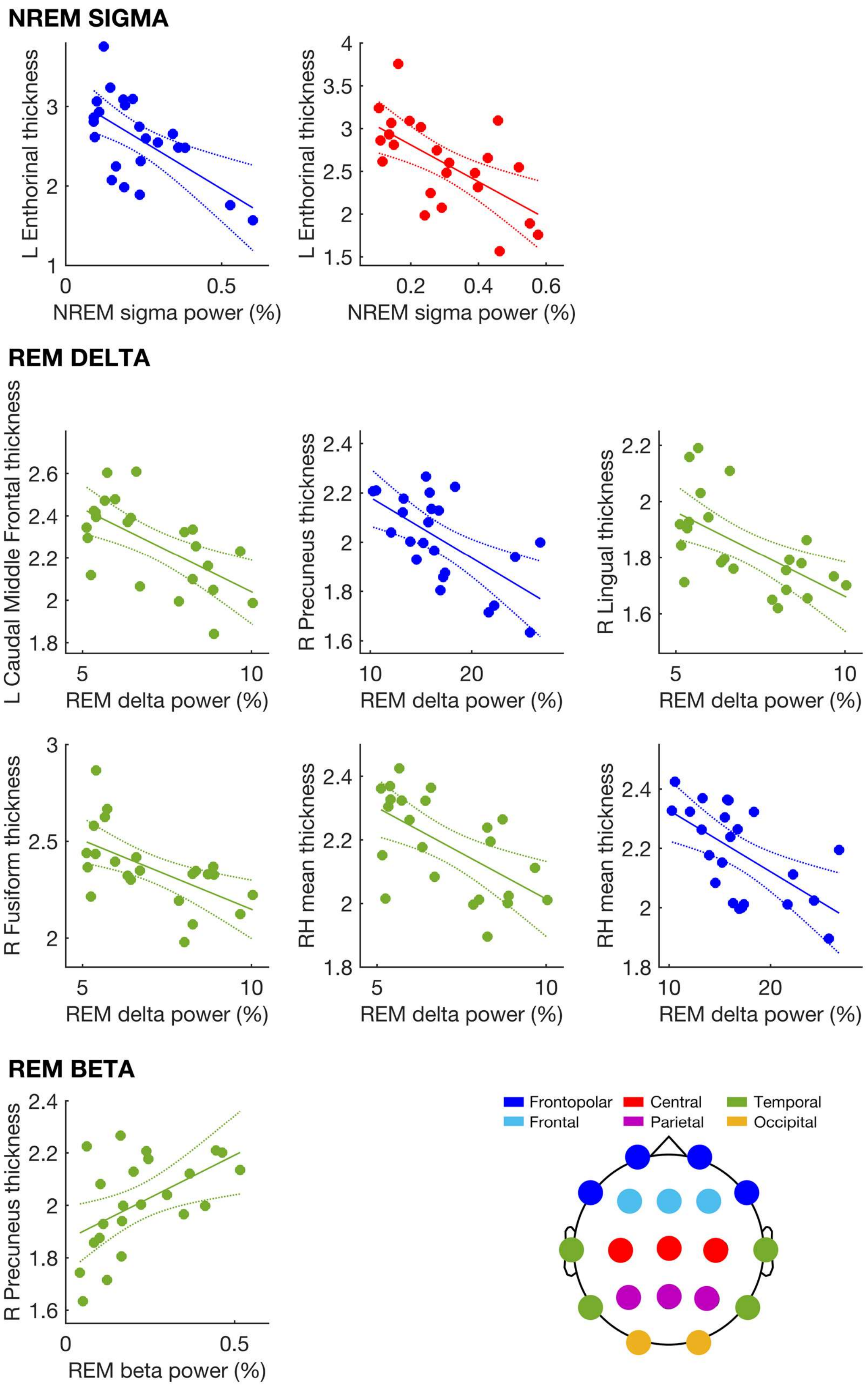

Figure S2. Correlations between cortical thickness and sleep EEG indexes. Cortical thickness (mm) vs. EEG power (%) scatterplots for cortical areas and sleep EEG indexes showing a significant relationship after FDR correction but excluded from Figure 2 in the main text.

Tables

Table S1. Results from the control analysis with partial correlations (partial r coefficients and corresponding p values) controlling for age and sex on cortical thickness and relevant EEG indexes of NREM and REM sleep showing significant associations in the principal analysis.

RM Cortical areas	Side	sleep index	EEG cluster	Partial r	p
Caudal middle frontal	L	REM Delta	Temporal	-0.61	0.0034
	R	REM Delta	Temporal	-0.64	0.0020
Entorhinal	L	NREM sigma	Frontopolar	-0.62	0.0028
	L	NREM sigma	Frontal	-0.67	0.00090
	L	NREM sigma	Central	-0.62	0.0027
Fusiform	R	REM Delta	Frontopolar	-0.71	0.00029
	R	REM Delta	Temporal	-0.53	0.014
Lingual	R	REM Delta	Frontopolar	-0.65	0.0016
	R	REM Delta	Temporal	-0.55	0.0098
Precuneus	R	NREM sigma	Parietal	0.53	0.013
	R	REM delta	Frontopolar	-0.69	0.00049
	R	REM delta	Temporal	-0.68	0.00069
	R	REM beta	Temporal	0.51	0.017
	R	REM beta	Occipital	0.51	0.017
Superior parietal	R	REM delta	Temporal	-0.60	0.0039
Mean thickness	R	REM delta	Frontopolar	-0.63	0.0024
	R	REM delta	Temporal	-0.61	0.0034

Abbreviations: MR, magnetic resonance; L, left hemisphere; R, right hemisphere; NREM, non-rapid eye movement sleep; REM, rapid eye movement sleep.
